# Rapid Molecular Response to Dasatinib in a Pediatric Relapsed Acute Lymphoblastic Leukemia With *NCOR1-LYN* Fusion

**DOI:** 10.3389/fonc.2020.00359

**Published:** 2020-03-20

**Authors:** Hai-Ping Dai, Jia Yin, Zheng Li, Chun-Xiao Yang, Tin Cao, Ping Chen, Yun-Hui Zong, Ming-Qing Zhu, Xia-Ming Zhu, Sheng Xiao, De-Pei Wu, Xiao-Wen Tang

**Affiliations:** ^1^National Clinical Research Center for Hematologic Diseases, Jiangsu Institute of Hematology, The First Affiliated Hospital of Soochow University, Suzhou, China; ^2^Institute of Blood and Marrow Transplantation, Collaborative Innovation Center of Hematology, Soochow University, Suzhou, China; ^3^Collaborative Innovation Center of Hematology, Soochow University, Suzhou, China; ^4^Sano Suzhou Precision Medicine Co., Ltd., Suzhou, China; ^5^Department of Pathology, Harvard Medical School, Brigham and Women's Hospital, Boston, MA, United States

**Keywords:** *NCOR1-LYN*, fusion gene, dasatinib, ALL, pediatric

## Abstract

**Background:** Philadelphia chromosome-like acute lymphoblastic leukemia (Ph-like ALL) is associated with high rates of treatment failure and poor outcome. Activation of ABL/Src family kinases is found in ~10% of Ph-like ALL, which can be therapeutically targeted by tyrosine kinase inhibitors. LYN is a member of the ABL/Src-tyrosine kinase family. Somatic *LYN* rearrangements are found in 5 cases of hematopoietic malignancies so far, although none of them were treated with tyrosine kinase inhibitors.

**Case presentation:** A 6-year-old boy with relapsed B-ALL had no response to reinduction chemotherapy. He was then treated with the *ABL1* tyrosine kinase inhibitor dasatinib and achieved complete remission within 2 weeks. Haploidentical allogenic stem cell transplantation (allo-HSCT) was subsequently performed and maintenance therapy with dasatinib initiated 8 weeks post-transplantation. He has been in minimal residual disease negative remission for 10 months after allo-HSCT.

**Result:** His bone marrow karyotype showed a balanced translocation between chromosomes 8 and 17, leading to a *NCOR1-LYN* fusion gene confirmed with sequencing.

**Conclusion:** Although *LYN* overexpression is described in many AML and B-ALL patients, intragenic *LYN* rearrangement is a rare event. For the first time, we present evidence that dasatinib is effective in treating a pediatric B-ALL with *NCOR-LYN* fusion.

## Background

In spite of the excellent prognosis of pediatric B-ALL, disease relapse still reaches as high as 15–20% of these patients, which remains the major cause of leukemia-related death. High-risk B-ALL, such as Philadelphia chromosome-like acute lymphoblastic leukemia (Ph-like ALL), is associated with higher rates of treatment failure, elevated minimal residual disease (MRD) levels, early relapse, and poor outcome (1). Activation of ABL/Src family kinases or JAK family kinases resulted from chromosome rearrangements occur frequently in patients with such characters, which can be therapeutically targeted by ABL/Src or JAK inhibitors, respectively (2). LYN is a member of the ABL/Src-tyrosine kinase family. Intragenic *LYN* rearrangement has been reported in 5 cases of hematopoietic malignancies so far (3–8) (Table 1). Although *in vitro* studies showed that the ABL/Src inhibitors were capable of blocking LYN's kinase activities, their clinical efficacy in real patients remains unknown (9). Here, we report a pediatric relapsed B-ALL with a t(8;17)(q12;p11.2)/*NCOR1-LYN* fusion showing robust and rapid response to dasatinib monotherapy.

**Table 1:** Characteristics of the reported and the present cases with a LYN rearrangement.

**Cases**	**Age(year)/gender**	**Disease**	**Initial WBC counts**	**Karyotype**	**Fusion gene**	**Additional genetic changes**	**Relapse**	**TKI**	**Allo-HSCT**	**Clinical outcome**
Tanaka et al. (4)	21/male	PMF^*^	25.5 × 10^9^/L	46,XY,ins(12;8)(p13;q11q21)	*ETV6-LYN*	Unknown	No	Imatinib	Yes	Dead
Telford et al. (5)	46/male	MPN^*^	17.2 × 10^9^/L	46,XY,der(8)inv(q12.1q21.1)t (8;12)(q12.1;p13),der(12)t(8;12) (q12.1;p13)[2]^#^	*ETV6-LYN*	Unknown	No	No	No	Dead
Ma et al. (6)	41/male	AML	16.1 × 10^9^/L	47,XY,add(1)(p13), der(12)t(1;12)(p13;p12),+mar [19]/46,XY[1]	*ETV6-LYN*	No	No	No	Yes	Unknown
Reshmi et al. (9)	Unknown	B-ALL	Unknown	Unknown	*GATAD2A*-*LYN*	Unknown	Unknown	Unknown	Unknown	Unknown
Yano et al. (7) and Imamura et al. (8)	8/female	B-ALL	293 × 10^9^/L	No metaphases	*NCOR1-LYN*	Deletion of *IKZF1, BTG1,CDK* *N2A/2B*	Yes	No	Yes	CR^*^
The present case	6/male	B-ALL	883 × 10^9^/L	46,XY,t(8;17)(q12;p11.2[10]/48, idem,+der(17)t(9;17),+22/46,XY[1]) [9]/46, XY[9]	*NCOR1-LYN*	Deletion of *IKZF1, CDKN2A*	Yes	Dasatinib	Yes	CMR^*^

* *CR: complete remission; CMR: complete molecular remission; MPN: myeloproliferative neoplasm; PMF: primary myelofibrosis*.

# *The intact karyotype was as: 46,XY,der(8)inv(q12.1q21.1)t(8;12)(q12.1;p13),der(12)t(8;12)(q12.1;p13)[2]/47,sl,+der(8)inv(8)t(8;12)[5]*.

*/48,sdl1,+der(8)inv(8)t(8;12)[2]/46,XY[2]*.

## Case Presentation

**Figure 1:**
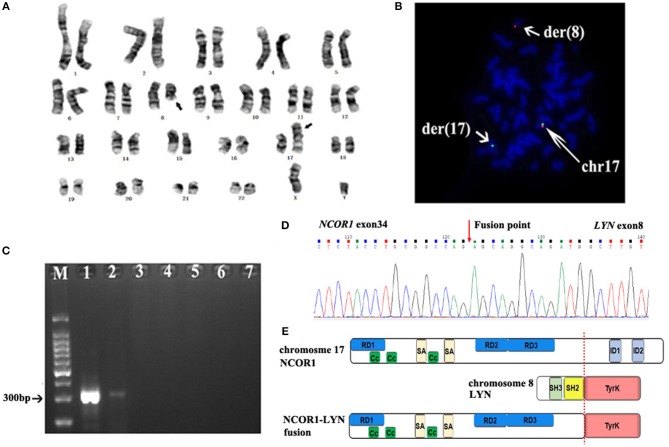
G-banding analysis of the BM sample at relapse, which showed the balanced translocation t(8;17)(q12;p11.2). **(B)** FISH with a chromosome 17 centromere probe (green) and a *TP53* probe (red) showed one of the *TP53* signals on the der (8) chromosome, suggesting that the chromosome 17p breakpoint is centromeric to *TP53* on 17p13. **(C)** RT-PCR showed various levels of *NCOR1-LYN* fusion transcript in bone marrow specimens. Lane 1: relapse sample; lane 2: 2 weeks after dasatinib; lane 3: 1 month post allo-HSCT; lane 4: 2 months post allo-HSCT; lane 5: 3 months post allo-HSCT; lane 6: 10 months post allo-HSCT; lane 7: negative control. **(D)** Sanger sequencing result of the *NCOR1-LYN* fusion gene, confirming a fusion between exon 34 of *NCOR1* and exon 8 of *LYN*. **(E)** Schematic representation of the predicted domain structure of the NCOR1-LYN fusion.

**Figure 2:**
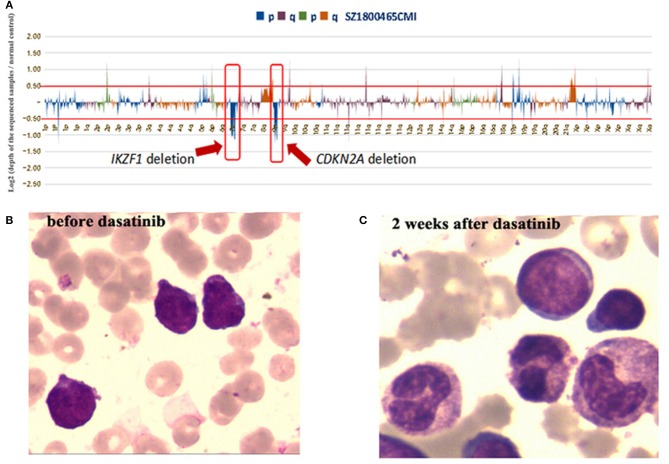
NGS analysis of the bone marrow sample at relapse showed deletions of CDKN2A and IKZF1. **(B,C)** Bone marrow smears before and after dasatinib therapy.

The patient presented with swollen gums in March 2015 in an outside hospital. A complete peripheral blood cell count showed leukocytes 883 × 10^9^/L, Hb 56g/L and platelets 41 × 10^9^/L. Bone marrow histology showed 97.6% of blasts, which were negative for myeloid peroxidase. Flow cytometry demonstrated 91.3% of blasts that were positive for CD10, CD19, CD22, and cyCD79a. Karyotype analysis of the bone marrow specimen found only 2 metaphases with normal 46, XY karyotype. Fluorescence *in situ* hybridization (FISH) studies were negative for *BCR/ABL1, ETV6/RUNX1* translocations and *KMT2A* (*MLL), MYC* and *PDGFRB* rearrangements. Result of multiplex PCR covering 41 fusion genes commonly detected in ALL was negative. A diagnosis of B-ALL was established. The patient was treated with daunorubicin (DNR), vincrinstine (VCR), PEG asparaginase (PEG-ASP) according to the Chinese Children's Cancer Group (CCCG)-2015-ALL protocol (10) and achieved complete hematological remission at the end of induction chemotherapy. Minimal residual disease (MRD) based on flow cytometry remained positive (≥1 × 10^−4^) during the subsequent chemotherapy. HSCT was not performed due to parents' concern on potential HSCT-related complications. The patient received 3 years' chemotherapy following the CCCG-2015-ALL protocol. Consolidation chemotherapy included 4 cycles of high-dose methotrexate (MTX), followed with 5 cycles of combined chemotherapy with dexamethasone (Dex), DNR, VCR, PEG-ASP, and cytarabine. Maintenance therapy comprised cycles of 6-mercaptopurine and MTX, which was completed in March 2018. Unfortunately, disease relapsed in September 2018 (42 months after the initial diagnosis), and he was admitted to our hospital. A complete peripheral blood cell count showed leukocytes 44.8 × 10^9^/L, Hb 64 g/L and platelets 99 × 10^9^/L. Blast counts of bone marrow were 71.5% by histology and 84.1% by flow cytometry, blasts were positive for CD10, CD19, CD22, CD38, and cyCD79a and negative for CD20. Chromosome analysis of the bone marrow specimen showed 46,XY,t(8;17)(q12;p11.2),9qh+[10]/48,idem,+der(17)t(8;17),+22[9]/46,XY,9qh+[1] (Figure 1A). Fluorescence *in situ* hybridization (FISH) analysis with a panel of FISH probes specific to Ph-like B-ALL, including *ABL1, ABL2, JAK2, CRLF2*, and *EPOR*, were all negative (data not shown). FISH with a chromosome 17 centromere probe and a *TP53* probe showed that one of the *TP53* signals was relocated to the der (8) chromosome, consistent with a chromosome 17 breakpoint which was centromeric to *TP53* (Figure 1B). Because *NCOR1* is located at 17p11.2, we assumed that the t(8;17)(q12;p11.2) led to *NCOR1/LYN* fusion. PCR with primers specific to *NCOR1* (5′ -CGTACAACTCTGCTTCCATGTCTC-3′) and *LYN* (5′-GCCACCTTGGTACTGTTGTTATAGTAAC-3′) showed a sharp band, with a size consistent with the *NCOR1/LYN* fusion; while no such band was detected using placenta control RNA template (Figure 1C). Sanger sequencing of the PCR band confirmed that the *NCOR1* exon 34 was fused to *LYN* exon 8 (Figure 1D). In addition to the *NCOR-LYN* fusion, several gene mutations were also observed in a concurrent next-generation sequencing assay, *ARID1A* Ala41Val with a variant allele frequency (VAF) of 63.1%, *KRAS* Gly12Ala with VAF of 0.6%, *NRAS* Gln61His with VAF of 2.2%, *PAX5* Arg140Leu with VAF of 39.9% and *ZNF292* Asn1695del with VAF of 43.4% (data not shown), and deletion of *CDKN2A* and *IKZF1* (Figure 2A). Unfortunately, we are unable to determine whether these genomic changes, including the *NCOR-LYN* fusion, are also present in the diagnostic specimen, due to lack of sample.

The patient was initially treated with mitoxantrone, vincristine and Dex. After completion of chemotherapy, bone marrow morphology still showed 61.5% of blasts (Figure 2B). The patient was then treated with dasatinib (60 mg/m^2^, once daily) as monotherapy. Two weeks after dasatinib treatment, bone marrow aspirates showed only 1% of blasts by histology (Figure 2C) and 0.1% of blasts by flow cytometry. Karyotype analysis of the bone marrow was normal. RT-PCR assays showed significantly decreased fusion transcript (Figure 1C). Haploidentical HSCT was performed 80 days after dasatinib therapy. No symptoms of graft vs. host disease were observed. Therefore, dasatinib was started 8 weeks post allo-HSCT for prevention of relapse. He has been tolerated with dasatinib very well and remained in MRD negative remission for 10 months now post allo-HSCT, based on both flow cytometry and RT-PCR assays (Figure 1C). Timeline of the treatment was shown in Supplementary Figure 1.

## Discussion

*LYN*, located on 8q12.1, is highly expressed in hematopoietic cells and plays roles in B-cell signaling, mast cell degranulation and erythroid differentiation (11). Four major functional domains of LYN include Src Homology 2 (SH2), SH3, proline-rich hinge region (P), and tyrosine kinase domain. In its inactivation status, SH2 and SH3 bind to LYN's phosphorylated carboxyl terminus (Y508) and P region, respectively, to ensure an inactivation conformation. LYN is activated when Y508 is dephosphorylated or the intramolecular binding released by competitive interaction with SH2 and/or SH3 (12). NCOR1 plays important roles in cellular metabolism, cell proliferation and neural stem cell differentiation (13–15). NCOR1 interacts with nuclear hormone receptors and recruits multiple histone deacetylase enzymes including HDAC1-4 and HDAC7 to form inhibitory chromatin structures. Four major functional domains of NCOR1 include repression domains (RD), coiled-coil domains (CC), SANT-like domains (SA) (for HDAC binding) and interactive domains (ID) (for nuclear hormone receptor binding). NCOR1-LYN fusion contains the 5′*NCOR1*, including RD, SA, and CC domains, and the 3′ *LYN* with an intact protein kinase domain (Figure 1E). Because CC function as oligomerization domains for a wide variety of proteins and are capable of both homo-oligomerization and hetero-oligomerization (13), we propose an oncogenic model that NCOR1-LYN homo-dimerization, driven by the CC domains from NCOR1, leads to trans-autophosphorylation within the activation loop of LYN kinase domain and constitutive LYN signaling.

Although *LYN* is over-expressed in acute myeloid leukemia (AML) and Ph^+^-ALL, intragenic rearrangements involving *LYN* are rare, with only 5 cases reported so far (Table 1) (4–8). The fusion partners of *LYN* include *GATAD2A, ETV6*, and *NCOR. GATAD2A-LYN* was reported in a patient with B-ALL with no clinic details of the disease (9). *ETV6-LYN* was reported in 2 patients with MPN and 1 patient with *de novo* AML. All three cases were resistant to conventional chemotherapy, progressed rapidly and succumbed to diseases in a short period of time (4–6). One of these patients was treated with imatinib with no response (4), although dasatinib was not used in any of these cases. Interestingly, *ETV6-LYN* transfected murine Ba/F3 hematopoietic cells were sensitive to dasatinib but not imatinib (4). Yano et al. reported the first B-ALL case with a *NCOR1-LYN* fusion gene (7, 8). Thus, our patient here confirmed that the *NCOR1-LYN* fusion is a recurrent event in B-ALL. Both patients had the same breakpoints at intron 7 of *LYN* and intron 34 of *NCOR1*. *IKZF1* gene defects occur in about 70% of Ph positive and Ph-like ALL cases and mediate therapy resistance (16). Therefore, we consider that *IKZF1* deletion might contribute to chemoresistance of this patient. Also, deletions of *IKZF1* and *CDKN2A* were observed in both cases. Clinically, both were pediatric B-ALL with high WBC counts at diagnosis who relapsed after chemotherapy. The previous case didn't receive tyrosine kinase inhibitors and survived after allo-HSCT (Table 1). Together these studies suggest that the pediatric B-ALL with *NCOR-LYN* fusion may have similar oncogenic mechanisms and clinical course.

## Conclusion

In summary, we present the first case of B-ALL with *NCOR1-LYN* fusion who showed a quick and robust response to dasatinib. Whether or not leukemia with *LYN* overexpression, in the absence of *LYN* rearrangement, is responsive to dasatinib is probably worth further evaluation.

## Ethics Statement

The studies involving human participants were reviewed and approved by the Ethics Committee of the First Affiliated Hospital of Soochow University. Written informed consent to participate in this study was provided by the participants' legal guardian/next of kin. Written informed consent was obtained from the minor(s)' legal guardian/next of kin for the publication of any potentially identifiable images or data included in this article.

## Consent

Written informed consent was obtained from the the patients' legal guardians for publication of this case report and the accompanying images.

## Author Contributions

C-XY, TC, and SX designed and interpreted data of the genetic analysis. Y-HZ and M-QZ performed flowcytometry analysis. H-PD, JY, ZL, X-MZ, D-PW, and X-WT treated the patient. H-PD and SX wrote the manuscript. D-PW and X-WT revised the manuscript. All authors approved the final version of the manuscript.

### Conflict of Interest

C-XY, TC, PC, and Y-HZ are employed by the company Sano Suzhou Precision Medicine Co., Ltd. The remaining authors declare that the research was conducted in the absence of any commercial or financial relationships that could be construed as a potential conflict of interest.

## Supplementary Material

Supplementary Figure 1 Timeline of the treatment.

## References

[1] Roberts KG Gu Z Payne-Turner D McCastlain K Harvey RC Chen IM. High frequency and poor outcome of philadelphia chromosome-Like acute lymphoblastic leukemia in adults. J Clin Oncol. (2017) 35:394–401. 10.1200/JCO.2016.69.0073 27870571 PMC5455698

[2] Roberts KG Yang YL Payne-Turner D Lin W Files JK Dickerson K. Oncogenic role and therapeutic targeting of ABL-class and JAK-STAT activating kinase alterations in Ph-like ALL. Blood Adv. (2017) 1:1657–71. 10.1182/bloodadvances.2017011296 29296813 PMC5728345

[3] Tanaka H Takeuchi M Takeda Y Sakai S Abe D Ohwada C. Identification of a novel TEL-Lyn fusion gene in primary myelofibrosis. Leukemia. (2010) 24:197–200. 10.1038/leu.2009.167 19710703

[4] Telford N Alexander S McGinn OJ Williams M Wood KM Bloor A. Myeloproliferative neoplasm with eosinophilia and T-lymphoblastic lymphoma with ETV6-LYN gene fusion. Blood Cancer J. (2016) 6:e412. 10.1038/bcj.2016.11 27058227 PMC4855251

[5] Ma ESK Wan TSK Au CH Ho DN Ma SY Ng MHL Next-generation sequencing and molecular cytogenetic characterization of ETV6-LYN fusion due to chromosomes 1, 8 and 12 rearrangement in acute myeloid leukemia. Cancer Genet. (2017) 218–219:15–9. 10.1016/j.cancergen.2017.09.001 29153093

[6] Yano M Imamura T Asai D Kiyokawa N Nakabayashi K Matsumoto K. Identification of novel kinase fusion transcripts in paediatric B cell precursor acute lymphoblastic leukaemia with IKZF1 deletion. Br J Haematol. (2015) 171:813–7. 10.1111/bjh.13757 26404892

[7] Imamura T Kiyokawa N Kato M Imai C Okamoto Y Yano M. Characterization of pediatric Philadelphia-negative B-cell precursor acute lymphoblastic leukemia with kinase fusions in Japan. Blood Cancer J. (2016) 6:e419. 10.1038/bcj.2016.28 27176795 PMC4916297

[8] Reshmi SC Harvey RC Roberts KG Stonerock E Smith A Jenkins H. Targetable kinase gene fusions in high-risk B-ALL: a study from the Children's Oncology Group. Blood. (2017) 129:3352–61. 10.1182/blood-2016-12-758979 28408464 PMC5482101

[9] Roberts KG. Why and how to treat Ph-like ALL? Best Pract Res Clin Haematol. (2018) 31:351–6. 10.1016/j.beha.2018.09.003 30466746

[10] Cai J Yu J Zhu X Hu S Zhu Y Jiang H Chinese Children's Cancer Group childhood acute lymphoblastic leukaemia (ALL) 2015 study group (CCCG-ALL-2015). Treatment abandonment in childhood acute lymphoblastic leukaemia in China: a retrospective cohort study of the Chinese Children's Cancer Group. Arch Dis Child. (2019) 6:522–9. 10.1136/archdischild-2018-316181 30705079

[11] Ingley E. Functions of the Lyn tyrosine kinase in health and disease. Cell Commun Signal. (2012) 10:21. 10.1186/1478-811X-10-21 22805580 PMC3464935

[12] Xu Y Harder KW Huntington ND Hibbs ML Tarlinton DM. Lyn tyrosine kinase: accentuating the positive and the negative. Immunity. (2005) 22:9–18. 10.1016/S1074-7613(04)00381-4 15664155

[13] Wong MM Guo C Zhang J. Nuclear receptor corepressor complexes in cancer: mechanism, function and regulation. Am J Clin Exp Urol. (2014) 2:169–87. 25374920 PMC4219314

[14] Sun Z Feng D Fang B Mullican SE You SH Lim HW. Deacetylase-independent function of HDAC3 in transcription and metabolism requires nuclear receptor corepressor. Mol Cell. (2013) 52:769–82. 10.1016/j.molcel.2013.10.022 24268577 PMC3877208

[15] Hermanson O Jepsen K Rosenfeld MG. N-CoR controls differentiation of neural stem cells into astrocytes. Nature. (2002) 419:934–9. 10.1038/nature01156 12410313

[16] Marke R van Leeuwen FN Scheijen B. The many faces of IKZF1 in B-cell precursor acute lymphoblastic leukemia. Haematologica. (2018) 103:565–74. 10.3324/haematol.2017.185603 29519871 PMC5865415

