# Supplementary figures and images for: Rapid Molecular Response to Dasatinib in a Pediatric Relapsed Acute Lymphoblastic Leukemia With NCOR1-LYN Fusion
Source: Front Oncol. 2020 Mar 20;10:359. doi: 10.3389/fonc.2020.00359 (PMC7098965; doi:10.3389/fonc.2020.00359)

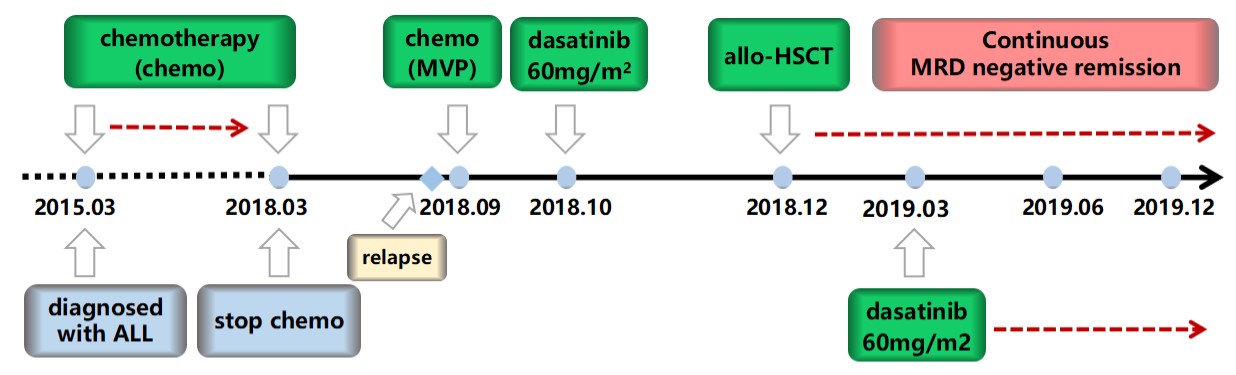

Supplement: Supplementary Figure 1 — Timeline of the treatment. [file Image_1.JPEG]
